# Nanomicellar Formulations Loaded with Histamine and Paclitaxel as a New Strategy to Improve Chemotherapy for Breast Cancer

**DOI:** 10.3390/ijms24043546

**Published:** 2023-02-10

**Authors:** Melisa B. Nicoud, Ignacio A. Ospital, Mónica A. Táquez Delgado, Jennifer Riedel, Pedro Fuentes, Ezequiel Bernabeu, Mara R. Rubinstein, Paolo Lauretta, Rocío Martínez Vivot, María de los Ángeles Aguilar, María J. Salgueiro, Daniela Speisky, Marcela A. Moretton, Diego A. Chiappetta, Vanina A. Medina

**Affiliations:** 1 Laboratorio de Biología Tumoral e Inflamación, Instituto de Investigaciones Biomédicas (BIOMED), Facultad de Ciencias Médicas, Pontificia Universidad Católica Argentina (UCA), Consejo Nacional de Investigaciones Científicas y Técnicas (CONICET), Buenos Aires 1107, Argentina; 2 Cátedra de Tecnología Farmacéutica I, Facultad de Farmacia y Bioquímica, Universidad de Buenos Aires, Buenos Aires 1113, Argentina; 3 Instituto de Tecnología Farmacéutica y Biofarmacia (InTecFyB), Universidad de Buenos Aires, Buenos Aires 1113, Argentina; 4 Laboratorio de Psiconeuroendocrinoinmunología, Instituto de Investigaciones Biomédicas (BIOMED), Facultad de Ciencias Médicas, Pontificia Universidad Católica Argentina (UCA), Consejo Nacional de Investigaciones Científicas y Técnicas (CONICET), Buenos Aires 1107, Argentina; 5 Laboratorio de Radioisótopos, Facultad de Farmacia y Bioquímica, Universidad de Buenos Aires, Buenos Aires 1113, Argentina; 6 Servicio de Patología, Hospital Británico de Buenos Aires, Buenos Aires 1280, Argentina

**Keywords:** nanomicellar formulation, drug delivery, histamine, triple negative breast cancer, paclitaxel, apoptosis, Soluplus^®^, polymeric micelles, combination therapy

## Abstract

Triple negative breast cancer (TNBC) is the most aggressive breast cancer subtype. Currently, paclitaxel (PTX) represents the first-line therapy for TNBC; however it presents a hydrophobic behavior and produces severe adverse effects. The aim of this work is to improve the therapeutic index of PTX through the design and characterization of novel nanomicellar polymeric formulations composed of a biocompatible copolymer Soluplus^®^ (S), surface-decorated with glucose (GS), and co-loaded either with histamine (HA, 5 mg/mL) and/or PTX (4 mg/mL). Their micellar size, evaluated by dynamic light scattering, showed a hydrodynamic diameter between 70 and 90 nm for loaded nanoformulations with a unimodal size distribution. Cytotoxicity and apoptosis assays were performed to assess their efficacy in vitro in human MDA-MB-231 and murine 4T1 TNBC cells rendering optimal antitumor efficacy in both cell lines for the nanoformulations with both drugs. In a model of TNBC developed in BALB/c mice with 4T1 cells, we found that all loaded micellar systems reduced tumor volume and that both HA and HA-PTX-loaded SG micelles reduced tumor weight and neovascularization compared with the empty micelles. We conclude that HA-PTX co-loaded micelles in addition to HA-loaded formulations present promising potential as nano-drug delivery systems for cancer chemotherapy.

## 1. Introduction

Breast cancer is the most frequent cancer diagnosis and the leading cause of cancer-related death in women worldwide. Globally, 2.3 million women have been diagnosed with breast cancer with a death toll of 685,000 only in 2020 [1,2]. It is a heterogeneous disease, and its molecular subtyping is principally based on the expression of the tumor’s estrogen receptor (ER), progesterone receptor (PR), and human epidermal growth factor receptor 2 (HER2). Targeting the aforementioned receptors represents an effective therapeutic option for a myriad of patients [2,3], except for the ones carrying a triple negative breast cancer (TNBC) subtype, lacking these receptors, and therefore a targeted treatment. TNBC accounts for 15–20% of the incident breast cancers and is an aggressive subtype, characterized by a short progression-free survival and poor prognosis [4,5], representing a major challenge in clinical oncology.

Chemotherapy represents the standard of care for TNBC. However, the response to this treatment is usually insufficient, followed by swift relapse and metastasis. As a single treatment or in combination, paclitaxel (PTX) is the first-line therapy for TNBC and one of the most widely used chemotherapeutic agents for several types of cancer. Its limited aqueous solubility is responsible for the drug’s severe adverse effects, habitually outweighing its clinical benefits and worsening the patient’s quality of life, limiting its clinical use. Promising nanotechnology-based delivery systems which encapsulate PTX and improve its therapeutic index have been under development for the past years, these include liposomes, solid lipid-based nanoparticles, polymeric nanoparticles, polymeric micelles, inorganic nanoparticles, and drug conjugates [6,7,8,9]. Polymeric micelles are one of the most auspicious emerging nanocarrier systems to improve the solubility and stability of hydrophobic drugs within the micelle core [8,10,11]. A novel nanocarrier for PTX delivery has been reported to enhance its performance in vitro, particularly, a glucose-functionalized mixed micelle formulation employing the co-polymeric solubilizer Soluplus^®^ and D-α-tocopheryl polyethylene glycol 1000 succinate (TPGS) in MCF-7 and MDA-MB-231 breast cancer cells when compared with the sole commercially available micellar-based PTX-nanoformulation (Genexol^®^) [7,12]. Additionally, albumin-bound PTX (nab-paclitaxel, Abraxane^®^) was the first PTX-based nanomedicine approved by the US Food and Drug Administration (FDA) for the treatment of metastatic breast cancer. Still, regardless of the clinical advantages described over the single molecule formulation, the clinical outcome of nanoformulations could be impaired due to drug resistance and long-term toxicity [6,8,13,14].

There is an overwhelming amount of evidence supporting the key role of histamine in breast cancer progression and in the response to cancer therapies. Administration of histamine in vivo reduced the growth of TNBC tumors developed in nude mice with MDA-MB-231 cells and in BALB/c mice with 4T1 cells, increasing median survival and tumor apoptosis [15,16]. Moreover, histamine has reduced cell proliferation in a dose-dependent manner, by inducing cell apoptosis and senescence at a micromolar concentration through the activation of the histamine H4 receptor (H4R) in human MDA-MB-231 and murine 4T1 TNBC cells [15,16,17]. Furthermore, the expression of the four histamine receptors subtypes have been described in human BC cell lines and tumor samples [18,19,20,21]. Preclinical data show that histamine improved conventional antitumor therapies response in different cancer types, supporting the rationale for the use of a combination therapy with histamine. In vivo, histamine heightened the antitumoral response to ionizing radiation and doxorubicin chemotherapy in TNBC experimental models. Interestingly, histamine protected highly radiosensitive tissues, including small intestine, salivary glands, and bone marrow from radiation-induced cytotoxic damage, and reduced hepatic and cardiotoxicity when combined with a chemotherapeutic treatment [22,23,24,25,26,27,28].

Based on this evidence, the aim of the present work was to improve the therapeutic index of PTX for the treatment of TNBC through the design and characterization of novel nanomicellar polymeric formulations co-loaded with histamine (HA) and PTX. The nanoformulations with both drugs showed optimal antitumor efficacy in TNBC cell lines, and in vivo studies demonstrated that both HA and PTX co-loaded micelles and HA-loaded formulations could be potential nano-drug delivery systems for cancer chemotherapy.

## 2. Results

### 2.1. Characterization of the Nanomicellar Formulations

**Figure 1:**
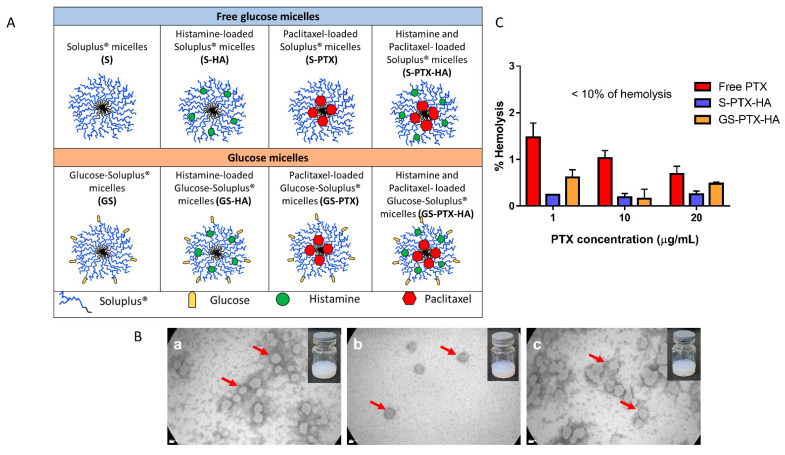
Characterization of nanomicellar polymeric formulations. (**A**) Hypothetical scheme. (**B**) TEM micrograph of (**a**) GS-HA, (**b**) GS-PTX, and (**c**) GS-PTX-HA micelles. Red arrows point out the polymeric micelles. Scale bar: 20 nm. Photo Inset: macroscopic aspect of the three nanoformulations. (**C**) Percentages of hemolysis of free PTX and PTX-HA co-loaded micellar systems after incubation with erythrocyte suspensions for 1 h at 37 °C. Error bars represent the mean ± SD (*n* = 3 independent experiments).

The main goal of the present study was the development of micellar nanoformulations co-loaded with HA and/or PTX either with or without glucose functional groups as a combined strategy to improve the therapeutic index of PTX in breast cancer therapy. Figure 1A shows a hypothetical scheme of the nanomicellar polymeric formulations.

First, we successfully developed a micellar nanoformulation co-encapsulated with both drugs employing 4 mg/mL of PTX and the highest concentration of HA (5 mg/mL) that could be incorporated within the polymeric micelles in order to obtain an adequate colloidal stability of the nanocarriers. Indeed, the colloidal dispersions remained translucent to the naked eye as depicted in Figure 1B and their colloidal stability was assessed by dynamic light scattering (DLS) assays as indicated in Section 2.2. This delivery system increased the apparent aqueous PTX solubility in more than 10000-fold, according to the drug intrinsic water-solubility [7].

Soluplus^®^ micelles (5% *w*/*v*, S) showed a narrow size distribution with a Z-ave value of 75.5 ± 0.2 nm, there was a slight micellar size increase to 77.9 ± 0.5 nm after PTX encapsulation, with only one size population. These results are in agreement with previous research based on mixed micelles of Soluplus^®^ (5% *w*/*v*) and TPGS loaded with PTX [29]. Interestingly, a similar trend was observed after the co-encapsulation of both PTX and HA within the polymeric micelles (78.3 ± 1.1 nm; PDI: 0.10) with a unimodal size distribution (Table 1, Figure S1).

The glycopolymer Soluplus^®^ (GS) described a slight size increment after PTX and HA encapsulation with narrow size distribution; Z-ave values were increased from 78.3 ± 1.1 nm (PDI: 0.10) to 87.2 ± 4.1 nm (PDI: 0.12) for S-PTX-HA micelles and GS-PTX-HA micelles, respectively (Table 1). A comparable trend was also observed for PTX-micelles and HA-micelles, before and after micellar glycosylation (Table 1, Figure S2) and similar results were reported for mixed polymeric micelles with Soluplus^®^ and glucose for PTX encapsulation [12].

The morphology of GS-HA, GS-PTX, and GS-PTX-HA micelles was visualized by TEM. All the nanoformulations presented spherical morphology with a unimodal size distribution in accordance with the size distribution data determined by DLS (Figure 1B). Similar results were observed previously for mixed Soluplus^®^-based micelles [29].

### 2.2. Stability of the Nanoformulations in Simulated Biological Fluid

Taking into consideration that polymeric micelles are dynamic colloidal systems [30], their stability after plasma dilution should be addressed for intravenous (i.v.) drug delivery. In the present study, micellar nanoformulations were diluted (1/33) in a Hank’s solution (pH 7.4) [31] and showed a unimodal size distribution with Z-average values between 140 and 320 nm, and PDI values between 0.06 and 0.35 (Table 2). Furthermore, the absence of unimers upon micellar dilution plus the absence of drug precipitation after sample dilution was an indication of the colloidal stability of the micellar nanocarrier. The drug concentration of the nanoformulations was quantified after the 1/33 dilution and approximately 98% of the drug concentration was maintained. Polymeric micelles have a core–shell nanostructure formed by self-assembly of amphiphilic polymers above critical micelle concentration (CMC). Their CMC represents a key parameter to estimate their physicochemical stability under dilution since they are dynamic colloidal systems. In this regard, both copolymers employed in the present study, Soluplus^®^ and Glucose-Soluplus^®^, have demonstrated a CMC of 0.002% *w*/*v* and 0.015% *w*/*v*, respectively, as determined by DLS in previous reports of our research group [12].Considering the mentioned CMC, the presence of intact polymeric micelles under dilution are guaranteed for both nanosystems, demonstrating that the present nanoformulations have an adequate colloidal stability for a potential i.v. administration.

### 2.3. In Vitro Hemolysis Assay

Since the nanoformulations are developed for a potential i.v. administration, the investigation of their hemolytic potential activity is fundamental. We compared the in vitro hemolytic performance of a PTX solution versus the nanoformulations loaded with both PTX and HA, in presence or absence of glucose. The maximum concentration assessed for PTX (20 µg/mL) was higher than its plasma therapeutic concentrations [32]. It was stressed that none of the formulations assayed showed in vitro hemolytic effect at the different PTX concentrations under research. For a PTX concentration of 20 µg/mL, a hemolytic percentage of 0.48 and 0.25 was observed for GS-PTX-HA micelles and the glucose-free counterpart, respectively (Figure 1C). Interestingly, the drug solution of PTX showed a higher hemolytic percentage than the micellar nanoformulations at the three drug concentrations evaluated (Figure 1C).

According to the international guidance for the in vitro hemolysis for pharmaceutical formulations [33], our nanoformulations are safe (non-hemolytic) for a potential i.v. administration to optimize breast cancer therapy.

### 2.4. In Vitro Antitumor Activity of Nanomicellar Formulations in TNBC Cells

#### 2.4.1. Cytotoxicity Assays

**Figure 2:**
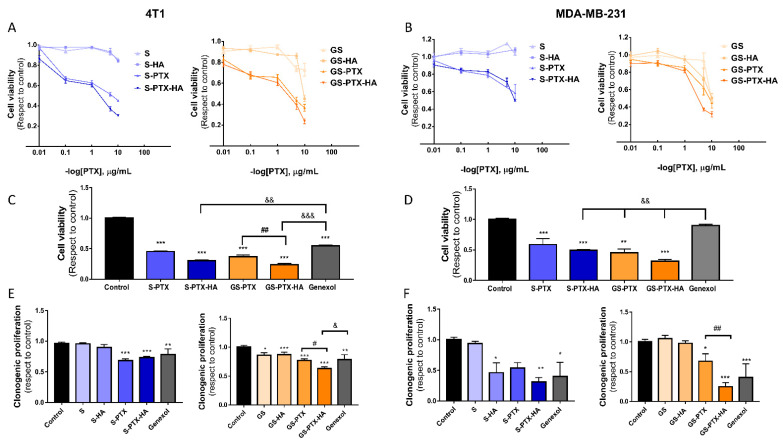
Cytotoxic effect of the micellar formulations. (**A**–**D**) Cell viability was evaluated by Celltiter-Blue assay after 72 h of treatment. (**C**,**D**) Cell viability at 10 μg/mL PTX and Genexol^®^ is shown. (**E**,**F**) *n* = 3–5 independent experiments performed in quintuplicate for each condition and cell line. Clonogenic proliferation was assessed in untreated (control) or treated cells with Soluplus^®^ micellar systems (PTX and Genexol^®^ 0.001 μg/mL) for 7 days. Error bars represent the mean ± SEM (*n* = 3 independent experiments performed in triplicate for each condition and cell line) (ANOVA and Tukey’s multiple comparisons test, * *p* < 0.05, ** *p* < 0.01, *** *p* < 0.001 vs. control; ^#^ *p* < 0.05, ^##^ *p* < 0.01; ^&^ *p* < 0.05, ^&&^ *p* < 0.01, ^&&&^ *p* < 0.001 vs. Genexol^®^).

Once nanosystems were characterized, in vitro studies were performed in 4T1 and MDA-MB-231 TNBC cell lines in order to evaluate their effect on cell viability. Glycosylated and non-glycosylated micelles loaded with PTX (S-PTX and GS-PTX) and co-loaded with PTX and HA (S-PTX-HA and GS-PTX-HA) reduced cell viability in a dose-dependent manner in both 4T1 (Figure 2A) and MDA-MB-231 cells (Figure 2B). GS-PTX-HA treatment produced the highest cytotoxic response compared with non-glycosylated micelles and GS-PTX at the highest concentration of PTX (10 μg/mL) evaluated in this assay. It is vital to highlight that GS-PTX-HA was even more effective in reducing cell viability than the commercial PTX-nanoformulation Genexol^®^ in both cell lines (Figure 2C,D).

In line with these results, a more sensitive growth assay to explore cell proliferation showed that all nanoformulations loaded with PTX (0.001 μg/mL) and the ones co-loaded with HA inhibited clonogenic proliferation in both cell lines. Again, only SG-HA-PTX formulation was capable of significantly reducing clonogenic proliferation compared with Genexol^®^ in 4T1 cells, and enhanced PTX cytotoxic effect in both cell lines at that low concentration (Figure 2E,F).

Considering the higher antiproliferative effects of the glycosylated nanoformulations compared with the glucose-free micelles and the commercially available nanoformulation Genexol^®^, we continued investigating the therapeutic potential of the PTX-HA-loaded glucose-decorated micelles. In line with these results, PTX (4 mg/mL) nanocarriers of glycosylated mixed micelles based in two biopolymers Soluplus^®^ and TPGS showed better in vitro antitumor performance in MCF-7 and MDA-MB-231 cells compared with Genexol^®^ [12]. Glucose accumulation in tumors further supports the rationale for the glucose functionalization of these PTX-HA-based drug delivery systems to preferentially target tumors [34,35,36].

#### 2.4.2. Apoptosis Assays

**Figure 3:**
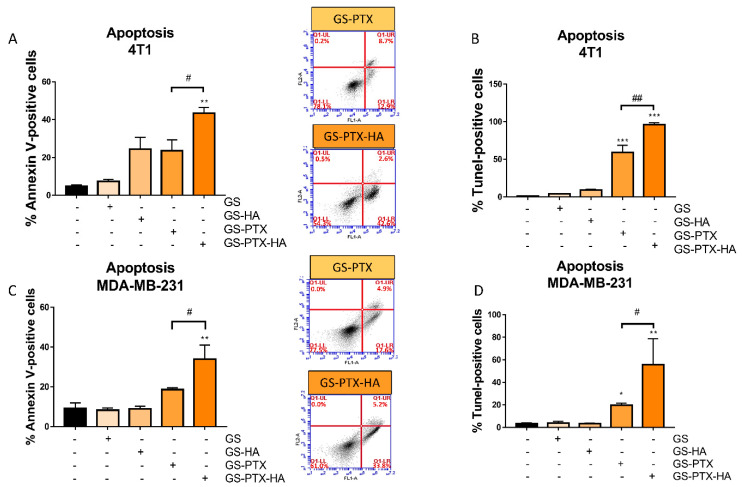
Modulation of apoptosis in 4T1 and MDA-MB-231 cells by nanomicellar formulations. (**A**,**B**) 4T1 and (**C**,**D**) MDA-MB-231 cells were treated with micellar systems (PTX 0.1 μg/mL) for 72 h. (**A**,**C**) The percentage of Annexin-V evaluated by flow cytometry. Annexin-V positive cells are shown in both right quadrants of dot plot. (**B**,**D**) TUNEL-positive cells are shown. Error bars represent the mean ± SEM (*n* = 3 independent experiments performed in triplicate) (ANOVA and Tukey’s multiple comparisons test, * *p* < 0.05, ** *p* < 0.01, *** *p* < 0.001 vs. control, ^#^ *p* < 0.05, ^##^ *p* < 0.01).

We investigated the impact of the glucose-decorated nanoformulations on cell apoptosis. The treatment of 4T1 and MDA-MB-231 cells with the PTX and HA co-loaded formulation (GS-PTX-HA) significantly increased the percentage of apoptotic cells evaluated by Annexin-V Assay and flow cytometry compared with the free-micellar system and the GS-PTX (Figure 3A,C). The apoptotic effect was further confirmed by the TUNEL assay, showing that HA potentiated the PTX-induced apoptosis in both cell lines (Figure 3B,D). Finally, the non-glycosylated S-PTX-HA formulation also increased the percentage of Annexin-V positive cells (Figure S3).

#### 2.4.3. Cell Migration Assays

**Figure 4:**
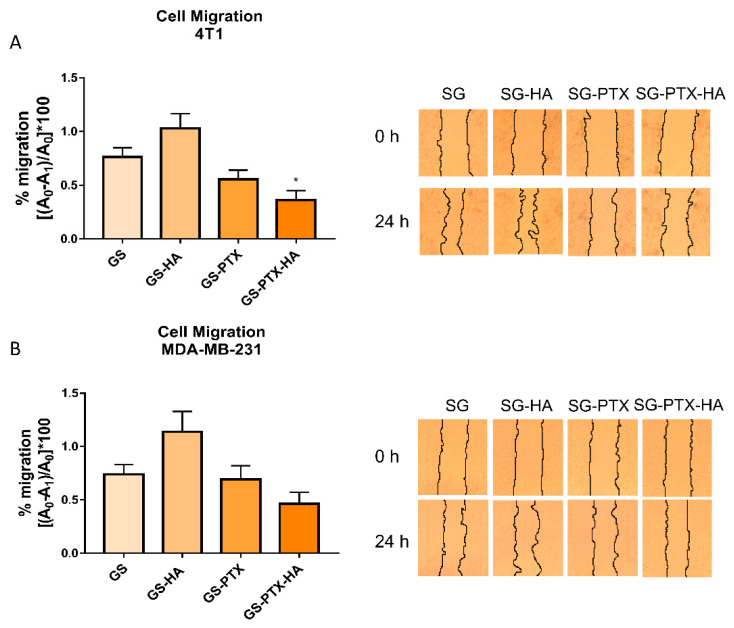
Modulation of cell migration in (**A**) 4T1 and (**B**) MDA-MB-231 cells by nanomicellar formulations. Cells were treated with micellar systems (PTX 0.01 μg/mL). The percentage of cell migration calculated [(A0 − A1)/A0] * 100 as with respect to control is shown. A0 is the initial area and A1 is the area after 24 h of incubation. Error bars represent the mean ± SEM (*n* = 3 independent experiments) (ANOVA and Tukey’s multiple comparisons test, * *p* < 0.05). Representative pictures are shown.

Cell migration is a key process during metastatic dissemination of cancer cells from the primary tumor to distant sites in the body, which is the leading cause of mortality in breast cancer patients [37]. To evaluate the effect of the novel nanoformulations, we performed the wound-healing assay, a classic and well-stablished method used to measure cell migration in vitro [38]. As a result, GS-HA-PTX co-loaded micelles (PTX 0.01 μg/mL) reduced cell migration after 24 h of treatment, an effect that was significant in 4T1 cells (Figure 4).

### 2.5. Cytotoxic Effect of Micellar Formulations in Murine Mammary Epithelial Cells

**Figure 5:**
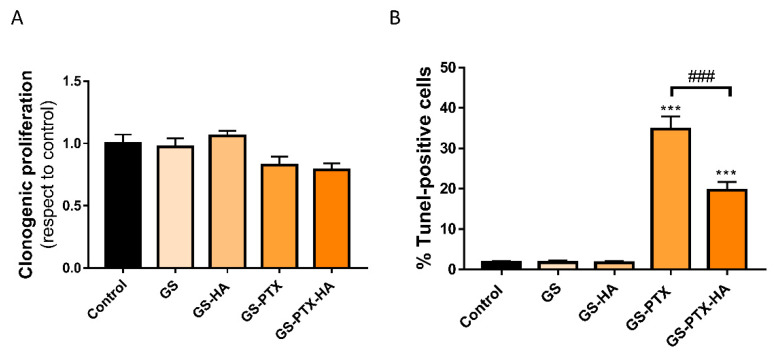
Effect of glycosylated nanosystems on the reproductive capacity and cell apoptosis of non-tumor NMuMG cells. (**A**) Clonogenic proliferation was evaluated (PTX 0.001 μg/mL). (**B**) TUNEL positive cells are depicted (PTX 0.1 μg/mL). Error bars represent the mean ± SEM. ANOVA and Tukey’s multiple comparisons test, *** *p* < 0.001 vs. control; ^###^ *p* < 0.001 SG-PTX-HA vs. SG-PTX).

NMuMG is an epithelial-like cell line that was isolated from murine mammary gland, and it was used as a non-breast cancer cell line to evaluate the selectivity of the cytotoxic effects of the micellar formulations. The results demonstrated that the treatment with GS-PTX and GS-PTX-HA did not affect clonogenic proliferation at the same concentration that reduced it in TNBC cells (PTX 0.001 μg/mL) (Figure 5A). Then again, although treatment with GS-PTX and GS-PTX-HA (PTX 0.1 μg/mL) increased the percentage of apoptotic cells, a reduced cell death was observed in GS-PTX-HA-treated compared with GS-PTX-treated cells in the TUNEL assay (Figure 5B). These results suggest a cytoprotective effect of histamine in non-tumor cells, which is in line with the previous studies that show a protective effect of histamine on different tissues, including the heart and submandibular glands, when combined with radiotherapy or chemotherapy [25,39].

### 2.6. In Vivo Antitumor Effect of Glycosylated Nanomicellar Formulations

**Figure 6:**
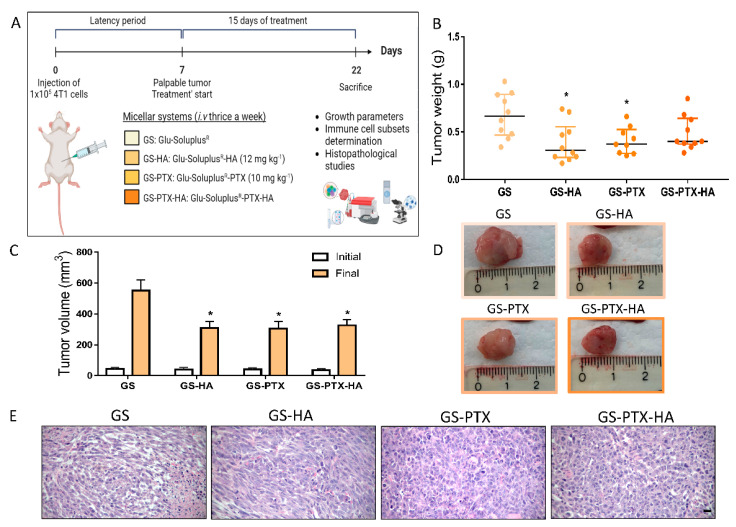
In vivo antitumor effects of glycosylated nanomicellar formulations. (**A**) Experimental design. Eight-week-old female mice with an average weight of 20–25 g were used for all experimental groups (*n* = 9–10). Figure created with BioRender.com. (**B**) Tumor weight. (**C**) Initial and final tumor volume. (**D**) Representative images of fresh tumors. (**E**) Representative images of tumor samples stained with H&E. ×400 original magnification. Scale bar = 20 µm. Scatter dot plots represent the median and interquartile range. ANOVA and Tukey’s multiple comparisons test, * *p* < 0.05 vs. GS.

**Figure 7:**
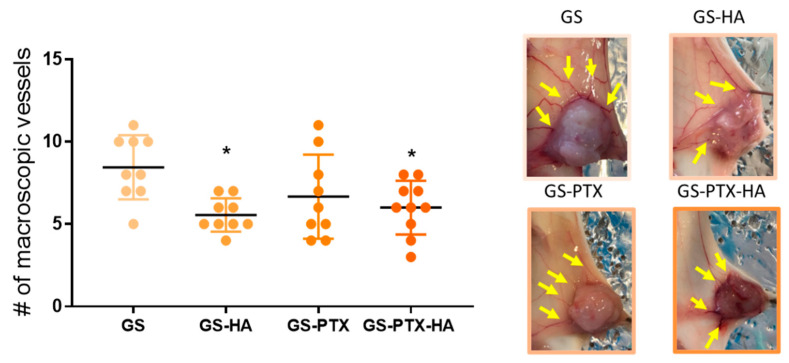
Number of macroscopic vessels. Scatter dot plots represent the median and interquartile range. ANOVA and Tukey’s multiple comparisons test, * *p* < 0.05 vs. GS. Representative images of the tumors are shown. Arrows indicate vessels.

In vivo studies were performed in a well-established TNBC experimental model. Approximately seven days after orthotopic inoculation of 4T1 TNBC cells, when tumors became palpable, animals were aleatory distributed into four different groups (Figure 6A), and were treated with GS, GS-HA (HA: 12 mg·kg^−1^), GS-PTX (PTX: 10 mg·kg^−1^), or GS-PTX-HA for 2 weeks, three times a week by i.v injection through the tail vein (Figure 6A). The study of the biological distribution of the GS micelles showed uptake in the tumors (Figure S4) and GS-HA and GS-PTX administration significantly reduced tumor weight while GS-PTX-HA treatment delineated a trend to reduce it (Figure 6B). All loaded nanoformulations reduced the tumor size and volume by the end of the experimental period (Figure 6C,D). The histological analysis of the hematoxylin and eosin (H&E)-stained tumors of the four experimental groups reported high grade infiltrating carcinoma, with solid sheets and marked nuclear pleomorphism, brisk mitotic activity, and large areas of necrosis (Figure 6E). These results were related to a significant decrease in the number of macroscopic tumor vessels in HA-loaded micelles (Figure 7). Neither any significant changes were observed in body weight at the end of the experiment (Figure S5A), nor any differences in the relative weights of heart, liver, and spleen (Figure S5B), or morphological abnormalities could be detected in these organs upon treatments, which suggest that formulations could be safely administered.

**Figure 8:**
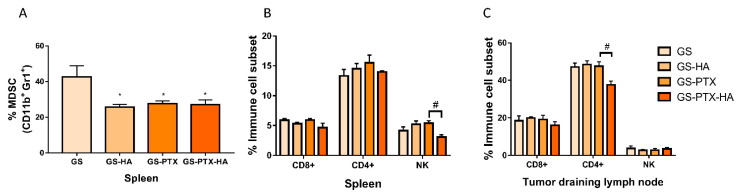
Immune cell subsets distribution. Cell suspensions from spleen and tumor draining lymph nodes-bearing mice were labelled with specific antibodies: (**A**) CD11b-PE and GR1-FITC: Myeloid-derived suppressor cells (MDSC), (**B**,**C**) CD4-FITC: T helper lymphocytes marker; CD8-PE: T cytotoxic lymphocytes marker and CD49-PE and CD3-FITC: Natural killer (NK) markers. Error bars represent the mean ± SD (ANOVA and Tukey’s multiple comparisons test, * *p* < 0.05 vs. GS, ^#^ *p* < 0.05 vs. GS-PTX).

An evaluation of the immune cell subsets in lymphoid organs was performed resulting in all loaded micellar systems-treated animals showing a reduction in the splenic myeloid-derived suppressor cells (MDSC) compared to the GS group (Figure 8A). In addition, treatment with GS-PTX-HA produced a decrease in the splenic percentage of natural killer (NK) cells compared with GS-PTX treatment, yet no changes were found in the percentages of T-helper (CD4+) and T-cytotoxic lymphocytes (CD8+) in this organ (Figure 8B). In tumor draining lymph nodes (TDLN), the proportion of CD4+ T cells was reduced while the percentage of CD8+ and NK cells remained unchanged in the GS-PTX-HA-treated animals when compared to the ones treated with GS-PTX (Figure 8C).

## 3. Discussion

In recent years, polymeric micelles prepared with biocompatible copolymers have improved the pharmacokinetic profile of hydrophobic chemotherapeutic drugs, including PTX, decreasing adverse effects and infusion times [40]. Additionally, the use of histamine in combination with traditional cancer therapies could deliver enhanced treatment results. A myriad of improved outcomes related to histamine administration have been previously reported such as: a synergistic anti-tumor effect when co-administered with doxorubicin (in vitro and in vivo), hepatic and cardiac doxorubicin toxicity amelioration in different pre-clinical experimental models [24,25], and histamine-functionalized block co-polymers presented as attractive delivery platforms for nucleic acids or doxorubicin, favoring endo-lysosomal escape of the carriers and their cargo into the cytoplasm [41,42,43,44,45]. In the present work, we developed micellar polymeric nanoformulations using the biocompatible polyvinyl caprolactam–polyvinyl acetate–polyethylene glycol graft copolymer (Soluplus^®^) co-loaded with histamine and PTX to improve this taxane-chemotherapy efficacy plus glycosylation was used as a potential strategy for active targeting and delivery improvement of the polymeric micelles to breast cancer cells [12,35,46].

We performed the physicochemical characterization and investigated in vivo and in vitro antitumor properties of the novel co-loaded nanomicelles in TNBC models. Glycosylation of HA and PTX-loaded formulations slightly increased the hydrodynamic diameter compared to the unglycosylated ones. All loaded nanoformulations showed a hydrodynamic diameter between 70 and 90 nm with a unimodal and narrow size distribution, dependent on the amphiphilic polymer and the encapsulated drug [30], and exhibited spherical morphology. Therefore, they present an optimal size that should be around 100 nm in hydrodynamic diameter for an adequate pharmacokinetic behavior. Although nanoparticles are defined as structures ranging from 1 to 100 nm in at least one dimension by The National Institutes of Health (NIH), their size is acceptable up to hundreds of nm for nanocarriers currently used in therapeutic applications. The aim of passive or active targeting ability of nanoparticles is to enrich the drug accumulation within a specific area, such as a tumor, while preventing the drug toxicity toward healthy tissues. Passive targeting depends on the characteristics of the tumor microenvironment and vessels, which are highly disorganized and have enlarged gap junctions between endothelial cells, resulting in enhanced permeability and retention (EPR) effect [47]. The EPR effect allows diffusion of structures of less than 400 nm in diameter, in accordance with the nanoscale micelles [8,48]. Active delivery is based on surface receptors upregulated on target tumor cells and could enhance cellular internalization [46]. We have chosen glucose as an active target considering that it is a key metabolic substrate with established roles in supporting breast cancer cell growth, hence, glucose moieties are widely employed as targeting ligands [35,46]. Moreover, the dilution of the nanoformulations in a simulated biological medium showed the absence of both unimers and drug precipitation indicating an adequate colloidal stability of the micellar nanocarrier for a potential systemic administration.

We evaluated the antitumor efficacy of the nanoformulations in vitro in human MDA-MB-231 and murine 4T1 TNBC cells. Previously, we have reported that a nanosystem of glycosylated Soluplus^®^ loaded with PTX (4 mg/mL) enhanced in vitro anti-glioma efficacy and improved in vitro antitumoral performance in MCF-7 and MDA-MB-231 versus Genexol^®^ [12,29]; in the present work, for both cell lines, HA co-treatment enhanced the PTX-induced reduction of cell viability and clonogenic proliferation and these effects were significant in the glycosylated micelles compared to the unglycosylated ones. Strikingly, GS-PTX-HA nanoformulation was even more effective in reducing TNBC cell proliferation than the FDA-approved micellar-based PTX-nanoformulation (Genexol^®^) [8]. These results are a buildup of our studies exclusively evaluating the therapeutic efficacy of the PTX and HA-loaded glucose-functionalized micelles. Furthermore, we have previously reported that HA synergized doxorubicin antitumor effect in MDA-MB-231 cells, augmenting DNA damage and cell apoptosis [24,25]. Other groups have also described a multifunctional targeting delivery system for doxorubicin that includes histamine in the micelle facilitated its anti-tumor efficacy in multidrug-resistant breast cancer cells, through the enhancement of doxorubicin release in the tumor site and reducing its heart’s uptake [44]. In addition, in vitro HA treatment potentiated gamma radiation-induced effects, increasing cell senescence and apoptosis of TNBC and also melanoma cell lines [16,39,49]. In this opportunity we show that the enhanced antiproliferative effect of GS-PTX-HA micelles compared to the GS-PTX ones was associated with an increased cell apoptosis produced by this combined nanoformulation, strengthening the idea that the effectiveness of the treatment is mediated by an increase in cell apoptosis.

Regardless of the intense research and the growing number of TNBC therapeutics, their effectiveness is usually transient due to the high risk of relapse associated with local or distant metastasis of this BC subtype [37]. We further investigated the effect of the glycosylated nanoformulations on cell migration, a key process for metastasis success. Interestingly, GS-PTX-HA micelles significantly reduced 4T1 cell migration compared to the empty micelle, suggesting that these nanoparticle-based carriers could improve metastatic breast cancer therapy. Further cell migration, invasion and metastasis studies are already being conducted to confirm this hypothesis.

Although anticancer nanomedicines have been investigated over three decades, less than ten nanoformulations have been approved for clinical therapy today due to multiple challenges, including the in vivo behavior of the nano-systems [8]. Auspiciously, in a murine model of TNBC developed in BALB/c mice with 4T1 cells, we found that all loaded SG micellar systems reduced tumor weight and size; however these effects were dramatically significant in the GS-HA and GS-PTX group. However, unlike the in vitro results, our in vivo evidence has not shown a better performance in tumor growth inhibition for the GS-HA-PTX group when compared to the GS-HA and GS-PTX-treated animals. Nevertheless, HA-loaded formulations (GS-HA and GS-PTX-HA) reduced neovascularization, a vital process for tumor growth, reinforcing the HA-induced inhibition of cell growth and antiangiogenic effect we had previously demonstrated in 4T1 tumors [16]. Furthermore, considering that HA dihydrochloride has been used as an adjuvant to IL-2 immunotherapy with proven clinical benefit in acute myeloid leukemia [50,51], it is important to point out that the GS-HA nanoformulation produced comparable antitumoral and antiangiogenic responses at the administered doses when compared to the chemotherapeutic-loaded micelles, highlighting the feasible therapeutic exploitation of this biogenic amine. The use of a system that involves an endogenous molecule provides a promising new approach for TNBC treatment with numerous advantages over chemotherapy in terms of short and long-term toxicity, and the therapeutic costs associated with the drug production and access to treatment. In addition to the reported direct effect of HA on TNBC cells through the activation of histamine receptors, HA is involved in immune-mediated responses that could affect tumor fate [16,51]. Strengthening this idea, we have described an immunomodulating effect of the GS-PTX-HA treatment, modifying subpopulations of immune cells including CD4^+^ T lymphocytes and NK cells in the tumor-draining lymph nodes and in the spleen, respectively. Considering the latest advances on immunotherapy of TNBC, it is worth mentioning that an FDA-approved combination of anti-programmed cell death-ligand 1 (PD-L1) immune checkpoint inhibitor (atezolizumab) plus nab-paclitaxel immunotherapy carries the potential of a more promising management of locally advanced or metastatic TNBC through tumor-specific and durable responses, but for a limited number of patients [52]. Furthermore, TME may hinder the efficacy of immunotherapy and chemotherapy, hence the potential immunomodulation effect of the GS-PTX-HA treatment may fulfill an encouraging role in advancing immunotherapeutic approaches and is definitely worth further exploring.

None of the formulations impaired body weight nor the relative weight of spleen, liver, or heart, and no morphological changes were determined in H&E-stained tissues, suggesting that the formulations did not present toxicity in terms of histopathology in the organs previously mentioned. A possible mild adverse effect observed was a local irritative reaction upon i.v. inoculation in PTX-loaded micelles but not in GS-HA group. Furthermore, the micellar nanoformulations presented a reduced hemolytic percentage compared to the drug solution of PTX proving to be safe, in terms of hemolytic capacity, for a potential i.v. administration to optimize breast cancer therapy. Therefore, all formulations were safe and well tolerated and showed efficacy in reducing tumor growth, providing a robust rationale for further investigation and development, including administered dose increments and new active targets, besides glucose, that could enhance the functionalization of the delivery.

We conclude that the developed polymeric micelles with biocompatible copolymer Soluplus^®^ surface decorated with glucose are promising and versatile nano-carriers of PTX and HA, and that HA-PTX co-loaded micelles in addition to HA-loaded formulations present potential as nano-drug delivery systems for cancer chemotherapy. Finally, despite the presented results are auspicious, more studies are needed to confirm the antitumor efficacy and evaluate the antimetastatic potential of these new nanotechnological strategies based on HA and PTX therapy, still signifying promising approaches for the treatment of TNBC.

## 4. Materials and Methods

### 4.1. Chemicals

Histamine (HA, 97.0%), d-gluconolactone (Glu; 1,2,3,4,5-pentahydroxycaproic acid d-lactone, MW = 178.14 g/mol), and tin(II) 2-ethylhexanoate (Sn(Oct)2, 95%) were purchased from Sigma–Aldrich (Buenos Aires, Argentina). Paclitaxel (PTX, 99.9%) was purchased from RhenochemAG (Basel, Switzerland) and Genexol^®^ (Gx, monomethoxy-poly(ethylene glycol)-b-poly(d,l-lactide)), polyvinyl caprolactam–polyvinylacetate–PEG (Soluplus, MW 120.000 g/mol) were acquired from BASF (Buenos Aires, Argentina).

### 4.2. Glycosylation of Soluplus^®^ Copolymer

**Figure 9:**
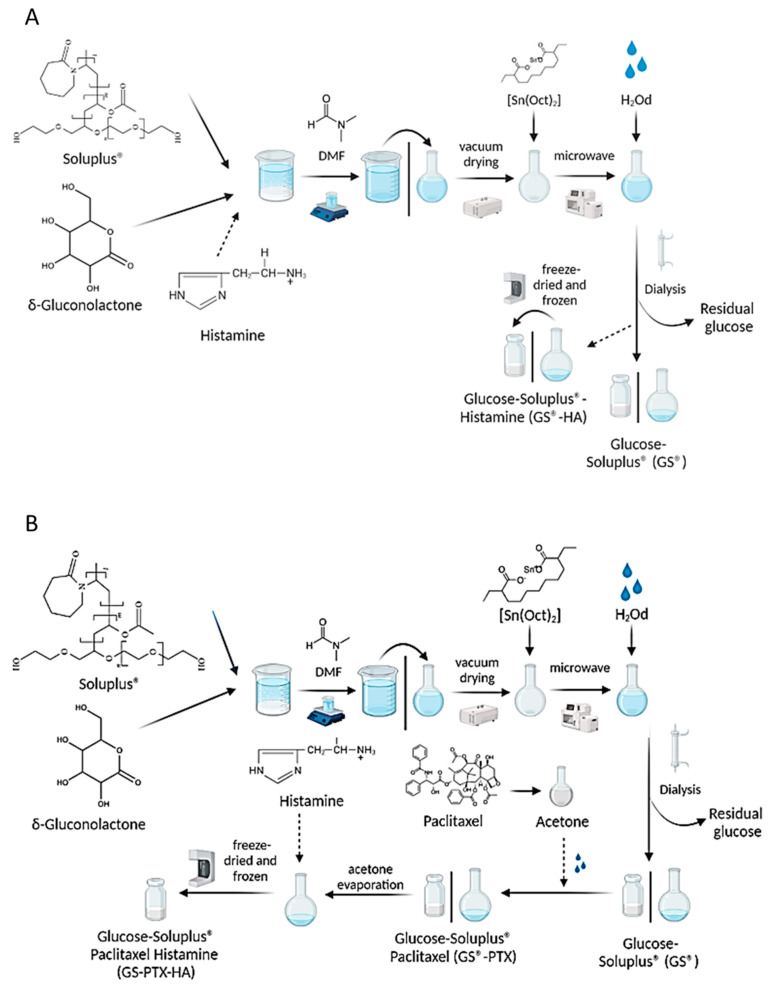
Schematic representation of Glucose-Soluplus^®^ micelles synthesis. (**A**) The arrows indicate the direction of the process, from the initial mixture of Soluplus^®^ and δ-gluconolactone to the final obtaining of freeze-dried glycosylated micelles (GS 5% *w*/*v*). The dotted arrows represent the possible addition of histamine in the synthesis to obtain histamine-glycosylated micelles (GS-HA 5 mg/mL). (**B**) The dotted arrows represent the possible addition of paclitaxel with or without further addition of histamine in the synthesis to obtain GS-PTX (4 mg/mL) and GS-PTX-HA (4 mg/mL: 5 mg/mL) micelles. References: DMF (dimethylformamide); Sn(oct)2 (tin octoate); H_2_Od (deionized water). Figure created with BioRender.com (accessed on 29 November 2022).

The glycosylation of the copolymer was assessed by a ring-opening reaction [29]. Initially, the copolymer (5 g) and gluconolactone (42.7 mg) were dissolved in dimethylformamide and the mixture was dried under vacuum for 3 h in a glycerol bath. Later, an aliquot of the catalyst (Sn(Oct)2 13.5 μL) was added to the reaction mixture. Afterwards, the sample was heated using microwave radiation for 15 min using power level 2 (160 W) for 1 min and power level 1 (80 W) for the additional 14 min (Microwave oven Whirlpool^®^, WMD20SB, 2450 MHz, potency 800 W, Buenos Aires, Argentina). Thereafter, the reaction mixture was dialyzed (3 d, Spectra/Por^®^ Dialysis Membrane, molecular weight cut off = 3500, nominal flat width 45 mm, Waltham, MA, USA), previously diluted with distilled water (10 mL). Finally, glycosylated copolymer was freeze-dried (freezing temperature −20 °C, 48 h, FIC-L05, FIC, Scientific Instrumental Manufacturing, Buenos Aires, Argentina) before use. The glycosylated derivative was denoted as GS (Figure 9A).

### 4.3. Preparation of Soluplus^®^ Micelles and Drug Encapsulation

Soluplus^®^ micelles (S) were prepared by the copolymer dispersion in distilled water (5% *w*/*v*) under magnetic stirring (50 RPM) over 1 h at room temperature, micellar dispersions were stored overnight before use. A similar procedure was performed for the preparation of SG micelles (5% *w*/*v*).

A solvent-diffusion technique was employed to encapsulate PTX within the polymeric micelles, in absence (S-PTX) and presence of glucose (GS-PTX) [12]. Briefly, PTX was dissolved in acetone (30 mg/mL) under sonication (5 min, Digital Ultrasonic Cleaner, PS-10A 50/60 Hz, Shenzhen, China, 25 °C); the PTX organic solution (1.33 mL) was then added under magnetic stirring (50 RPM, 4 h) drop by drop (programmable syringe infusion pump, PC11UB, APEMA, Buenos Aires, Argentina) into the micellar dispersion (10 mL) until complete acetone evaporation. Then, the volume of the PTX-loaded micellar systems was adjusted with distilled water to 10 mL in a volumetric flask. Samples were filtered (0.45 μm, acetate cellulose filters, Microclar, Buenos Aires, Argentina) in order to remove the un-dissolved drug. Finally, S-PTX and GS-PTX micelles were freeze-dried (freezing temperature −20 °C, 48 h, FIC-L05, FIC, Scientific Instrumental Manufacturing, Argentina) before use (Figure 9B).

For encapsulation within the polymeric micelles, 50 mg of HA was incorporated to the aqueous micellar dispersion (10 mL) with (GS-HA) and without glucose (S-HA) under magnetic stirring (50 RPM) for 30 min, the dispersion was filtered (0.45 μm, acetate cellulose filters, Microclar, Argentina), and the micelles were lyophilized (freezing temperature −20 °C, 48 h, FIC-L05, FIC, Scientific Instrumental Manufacturing, Argentina) before use (Figure 9A).

For PTX and HA co-encapsulation, with (GS-PTX-HA) and without glucose (S-PTX-HA), a combination of the previously described techniques was utilized. First, polymeric micelles were loaded with PTX and afterwards HA was incorporated to the PTX-loaded micellar dispersions. Finally, samples were freeze-dried before use (Figure 9B). Drug-free polymeric micelles were used as controls.

Micellar content of PTX and HA was determined by reverse phase high liquid chromatography (RP-HPLC). For PTX quantification, a reverse phase C18 column (Fluophase PFP, 4.6 mm × 250 mm, 5 μm, Thermo, Waltham, MA, USA) with a mobile phase of acetonitrile:water (50:50, *v*/*v*) and an injection volume of 20 μL with a flow rate of 1 mL/min were used. The detection was performed at 227 nm (UV-Detector, Shimadzu SPD-10A, Japan) [53].

After their derivatization with dansyl chloride, concentrations of HA in the samples were determined using the HPLC method. The chromatographic system consisted of a reversed phase C18 column (3 mm × 100 mm, 3.5 µm, XBridge, Waters, Drinagh, Ireland) at 25 °C, SCL-10A pump, an SIL-10A autosampler, a CTO-10AS column oven, and an SPD-10A UV-detector (Shimadzu, Tokyo, Japan) set at 254 nm. The mobile phase consisted of acetonitrile/water (70:30 *v*/*v*). The flow rate was 1.0 mL/min and the injection volume was 20 µL [54].

### 4.4. Characterization of Soluplus^®^ Micelles

Dynamic light scattering (DLS, scattering angle of θ = 173° to the incident beam, Zetasizer Nano-ZSP, ZEN5600, Malvern Instruments, UK) was performed to determine micellar size, size distribution, and polydispersity index (PDI) of the nanoformulations. Samples were re-dispersed in distilled water (2 mL) and equilibrated at 25 °C prior to the DLS analysis and the hydrodynamic diameter (Dh), PDI, and zeta potential values were expressed as the average of three measurements ± S.D.

Nanoformulations with glucose morphology were investigated by electronic transmission electron microscopy (TEM, Philips CM-12 TEM apparatus, FEI Company, The Netherlands). Briefly, GS-HA, GS-PTX, and GS-PTX-HA freeze-dried samples were re-dispersed in distilled water (2 mL) and aliquots (5 μL) were negatively stained with uranyl acetate (2% *w*/*v*).

### 4.5. Physicochemical Stability of the Nanoformulations in Simulated Biological Fluid

Nanoformulation physicochemical stability was evaluated in vitro after their dilution with Hank’s solution, which has been previously employed to simulate biological fluids [55]. The Hank’s solution was obtained by dissolving NaCl 8.00 g, KCl 0.40 g, CaCl_2_ 0.14 g, NaHCO_3_ 0.35 g, MgSO_4_·7H_2_O 0.06 g, MgCl_2_·6H_2_O 0.10 g, Na_2_HPO_4_·12H_2_O 0.06 g, KH_2_PO_4_ 0.06 g, and D-glucose 1.00 g in 1000 mL of distilled water [56]. To simulate the dilution process after i.v. administration, freeze-dried samples were re-dispersed in distilled water (1 mL) and they were diluted (1/33) in Hank’s solution (pH 7.4). The average micellar size and size distribution of the micellar systems was evaluated after sample dilution by DLS (scattering angle of θ = 173° to the incident beam, Zetasizer Nano-ZSP, ZEN5600, Malvern Instruments, Malvern, UK) at 37 °C. Results were expressed as the average of the three measurements ± S.D.

### 4.6. Cell Culture

The following cell lines were cultured in Roswell Park Memorial Institute Medium (RPMI) supplemented with 10% (*v*/*v*) FBS, 0.3 g L^−1^ glutamine, 100 µg mL^−1^ streptomycin, and 100 U mL^−1^ penicillin (all from Gibco BRL, Grand Island, NY, USA): triple negative breast cancer cell lines 4T1 (ATCC CRL-2539, murine) and MDA-MB-231 (CRM-HTB-26™, human). NMuMG murine mammary epithelial cell line (ATCC CRL-1636) was kindly provided by Dr. Roguin (School of Pharmacy and Biochemistry, University of Buenos Aires) and cultured in DMEM supplemented with 10% FBS and antibiotics. Cells were maintained at 37 °C in a humidified atmosphere containing 5% CO_2_.

### 4.7. Cell Proliferation Assays

The clonogenic assay is a gold standard method to estimate the cell reproductive capacity, and is considered the most sensitive cell growth assay [57]. Cells were seeded in 12-well plates (700 cells per well for 4T1 cells, and 1000 cells per well for MDA-MB-231 cells) and were treated or left untreated. After 7 days of incubation, cells were fixed with 10% (*v*/*v*) formaldehyde in PBS (Sigma Chemical Co., St. Louis, MO, USA) and stained with hematoxylin. The clonogenic proliferation was evaluated by counting the number of colonies, each colony was defined when counting 50 cells or more, and the result was expressed as the number of colonies on the treated wells relative to the untreated ones. Different concentrations (PTX 0.001–1 μg/mL) were investigated to determine the optimal. The lowest concentration of PTX that produced a significant effect on the clonogenic proliferation was employed (PTX 0.001 μg/mL).

Cell viability was determined by the fluorometric resazurin reduction method (CellTiter-Blue; Promega, Madison, WI, USA). Briefly, 2.5–5 × 10^5^ cells/mL were seeded at a final volume of 0.1 mL in 96-well flat-bottom microtiter plates and were treated or left untreated for 72 h in complete medium as indicated in the results section. Fluorescence was determined in a BMG Labtech NOVOstar MicroPlate Reader (Germany).

### 4.8. Apoptosis Determinations

#### 4.8.1. Annexin V Assay

Apoptotic cells exposure of phosphatidylserine was detected by flow cytometry after staining the cells with Annexin V-FITC (BD Biosciences, San José, CA, USA), and propidium iodide (50 µg/mL) according to the manufacturer’s instructions. Briefly, cells were seeded into 12-well plates (2.5 × 10^4^ cells/well) and were treated or left untreated (Control) as indicated in the results section for 72 h in complete medium. Cells were harvested, centrifuged, resuspended in Annexin buffer (1X) and labeled with Annexin V and propidium iodide. Double fluorescence of the cells was immediately measured by flow cytometry (BD Accuri C6, BDB). Data were analyzed using BD Accuri C6 software (BDB).

#### 4.8.2. TUNEL Assay

Cells were cultured on glass coverslips into 12-well plates for 24 h and were treated or left untreated (Control) for 72 h in complete medium as indicated in the results section. Cells were washed, fixed, and the fragmented DNA was detected using In Situ Cell Death Detection Kit (ROCHE, Basel, Switzerland) according to the manufacturer’s instructions. Cells were visualized using Primovert Carl Zeiss microscope (Germany). At least 500 cells were scored for each determination. The concentration assessed for apoptosis analysis (PTX 0.1 μg/mL) was in agreement with the previous reports [58].

### 4.9. Wound Assay

MDA-MB-231 (1–1.2 × 10^5^) and 4T1 (6 × 10^4^) cells were seeded in 6- and 12-well plates, respectively. A wound was made with a p200 pipette tip once the monolayer became confluent, and the debris were removed from the plates with several serum-free medium washes. Cells were left untreated or treated in serum-free medium as shown in the results section. Finally, photographs were taken at time 0 and 24 h after the wound using Primovert Carl Zeiss microscope (Germany). The percentage of migration was expressed as % migration: [(A0 − A1)/A0] * 100. Where A0 is the area covered by cells at time 0 of the wound and A1 the area 24 h after the wound. The concentration assessed for cell migration (PTX 0.01 μg/mL) was the lowest that produced a significant effect and was in agreement with the previous reports [59].

### 4.10. Breast Cancer Model

Female BALB/c mice were bred and kept in ventilated cages at our animal health care facility at 22 to 24 °C and 50% to 60% humidity on a 12 h light/dark cycle with food and water available ad libitum. About 6–8-week-old animals with an average weight of 20–25 g were used. All animal protocols were in accordance with the recommendations from the National Institute of Health Guide for the Care and Use of Laboratory Animals (NIH Publications No. 8023) and the Guidelines for the welfare and use of animals in cancer research. The BIOMED Institutional Committee for the Care and Use of Laboratory Animals reviewed and approved all the procedures that were in accordance with the ARRIVE guidelines for reporting experiments involving animals (CICUAL, approved protocol number 003/2021).

To generate solid tumors, 1 × 10^5^ syngeneic 4T1 cells in serum-free PBS were inoculated orthotopically in the abdominal mammary gland of the mice, as described previously [60,61] Tumor length and width were measured every 2 days by using callipers, and tumor volume was calculated as V = π/6 × length × width^2^.

The animals were randomized in four different groups once the tumors were palpable and were treated thrice a week with 100 µL of one of the following glycosylated nanoformulations by i.v. administration (tail vein) for 2 weeks: GS, GS-HA (HA: 12 mg kg^−1^ b.w.), GS-PTX (10 mg kg^−1^ b.w.) and GS-PTX-HA (Figure 6A). The PTX dose (10 mg kg^−1^) assessed is under the maximum tolerated in mice yet it is low enough to be useful for the evaluation of combination therapies based on nanotechnology strategies [58,62,63].

Mice were sacrificed by cervical dislocation, tissues were removed and weighted, and used for further analysis.

### 4.11. In Vitro Hemolytic Assay

Fresh blood from rats was collected in tubes containing 3.2% sodium citrate (1:9) and centrifuged at 3500 rpm for 10 min in order to separate the red blood cells (RBCs). Once collected, RCBs were washed with normal saline solution (NaCl 0.9% *w*/*v*), centrifuged (3500 rpm, 10 min), and the supernatant was removed. This procedure was repeated three times. Finally, RBCs were diluted with normal saline solution (final RBCs concentration: 10% *w*/*v*). This diluted RBCs suspension was used for the hemolytic study to determine the cytotoxic properties of the nanoformulations. The hemolysis assay was performed following a reported method [64] with minor modifications. Briefly, the erythrocyte suspension (750 µL) was incubated with 750 µL of (i) S-PTX-HA, (ii) GS-PTX-HA, or (iii) free PTX at 37 °C for 3 h. The final PTX concentrations were 1, 10, and 20 μg/mL. Saline solution was used as negative control (0% lysis), considering all the samples were prepared in saline solution, and distilled water was considered as positive control (100% lysis), employing a dilution (1:1) with the RCBs suspension.

Samples were incubated at 37 °C for 3 h by inversion (Mini Labroller LabNet Rotator, Edison, NJ, USA, 40 rpm) and centrifuged for 10 min at 3500 rpm (MiniSpin^®^ plus™, Eppendorf, Germany). Finally, hemoglobin absorbance was measured at 541 nm (8452A Diode Array Spectrophotometer, Hewlett Packard, Palo Alto, CA, USA), and the hemolysis percentage was calculated using the following equation:

Hemolysis (%) = (Abs sample − Abs negative)/(Abs positive − Abs negative) × 100(1)

where Abs negative and Abs positive correspond to the negative control and positive control, respectively. The CICUAL of the School of Pharmacy of the University of Buenos Aires (REDEC-2021-2792-E-UBA-DCT_FFYB) approved all the animal experiments and animal care. Assays were performed by triplicate and expressed as mean ± S.D.

### 4.12. Histopathological Analysis

To evaluate the histological characteristics of the tumors and tissues excised, they were fixed in 4% (*v*/*v*) formaldehyde in PBS (formalin buffer), paraffin embedded, and sliced into 3–4-μm-thick sections and stained with hematoxylin-eosin (H&E) staining (Biopur diagnostic, Buenos Aires, Argentina). Visualization was performed with an optical microscope (Axiolab Carl Zeiss, Jena, Germany) by an expert histopathologist (D.S.).

### 4.13. Flow Cytometry for Immunophenotyping

As previously described by Sterle et al., and Martinel Lamas et al., [60,61] standard staining methods were used for various cell surface markers from single-cell suspensions obtained from tumor-draining lymph nodes (TDLN) and spleens. The fluorochrome-conjugated antibodies used in this study were: FITC rat anti-mouse CD3 (T lymphocytes marker, Cat. #/N° Lot: 553126/6202975); FITC rat anti-mouse CD4 (T helper lymphocytes marker, Cat. #/N° Lot: 557303/6032902); PE rat anti-mouse CD8a (T cytotoxic lymphocytes marker, Cat. #/N° Lot: 565410/6063689); PE rat anti-mouse CD49b (NK cells marker, Cat. #/N° Lot: 561066/7271756); FITC rat anti-mouse Ly-6G and Ly6C (myeloid differentiation antigen GR-1 marker, Cat. #/N° Lot: 553126/6202975); PE rat anti-mouse CD11b (myeloid cell marker, Cat. #/N° Lot: 560408/6190826). Samples were run on a BD Accuri C6 flow cytometer (BDB), and data were analyzed by using the BD Accuri C6 software (BDB).

### 4.14. Statistical Analyses

The results are presented as the mean ± standard error of the mean (SEM), the mean ± standard error (SD), or the median and interquartile range, as indicated in the legends. Sample distributions were assessed for normality using the Shapiro–Wilk normality test. One-way ANOVA followed by Tukey’s multiple comparison test was used for comparisons between more than two groups. All statistical analyses were performed with GraphPad Prism version 7.00 (San Diego, CA, USA).

## Figures and Tables

**Table 1:** Micellar size and size distribution (PDI) of free and drug-loaded Soluplus^®^ micelles (5% *w*/*v*), in presence or absence of glucose, at 25 °C.

Samples	Histamine (5 mg/mL)	Paclitaxel (4 mg/mL)	Size ^1^	
			Z-Ave (nm) (±S.D.)	PDI (±S.D.)
Soluplus^®^ micelles	-	-	75.5 (0.2)	0.09 (0.01)
	√	-	77.9 (0.5)	0.09 (0.01)
	-	√	76.2 (0.9)	0.09 (0.01)
	√	√	78.3 (1.1)	0.10 (0.01)
Glucose-Soluplus^®^ micelles	-	-	75.7 (0.5)	0.11 (0.01)
	√	-	83.8 (0.6)	0.11 (0.01)
	-	√	79.9 (1.7)	0.19 (0.01)
	√	√	87.2 (4.1)	0.12 (0.01)

^1^ Data are expressed as mean ± S.D. (*n* = 3).

**Table 2:** Size and size distribution of nanosystems diluted 1/33 in a Hank’s solution (pH 7.4) to simulate the dilution process after i.v., administration measured by DLS at 37 °C.

Samples	Histamine (5 mg/mL)	Paclitaxel (4 mg/mL)	Size ^1^	
			Z-Ave (nm) (±S.D.)	PDI (±S.D.)
Soluplus^®^ Micelles (5% *w*/*v*)	√	-	180.8 (2.7)	0.06 (0.01)
	-	√	138.8 (8.6)	0.13 (0.01)
	√	√	197.6 (4.3)	0.13 (0.01)
Glucose-Soluplus^®^ micelles (5% *w*/*v*)	√	-	191.6 (1.6)	0.19 (0.02)
	-	√	155.1 (6.3)	0.23 (0.01)
	√	√	321.3 (3.8)	0.35 (0.04)

^1^ Data are expressed as mean ± S.D. (*n* = 3).

## Data Availability

The data that support the findings of this study are available from the corresponding author upon reasonable request.

## Supplementary Materials

The following supporting information can be downloaded at: https://www.mdpi.com/article/10.3390/ijms24043546/s1.
