# Supplementary material for: Nanomicellar Formulations Loaded with Histamine and Paclitaxel as a New Strategy to Improve Chemotherapy for Breast Cancer
Source: Int J Mol Sci. 2023 Feb 10;24(4):3546. doi: 10.3390/ijms24043546 (PMC9959774; doi:10.3390/ijms24043546)
Supplement: Supplementary file 1 [file ijms-24-03546-s001.zip › ijms-2135518-supplementary.pdf]

Figure S1

A

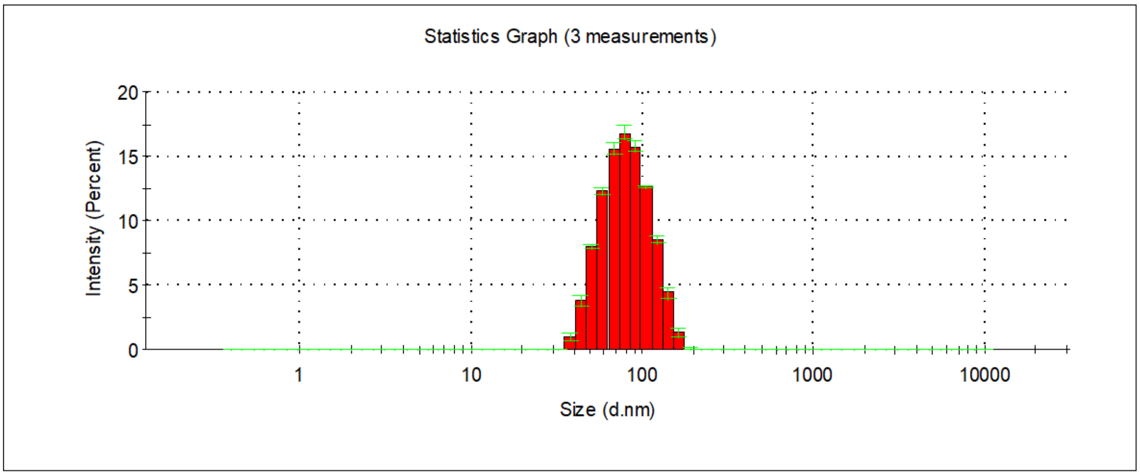

B

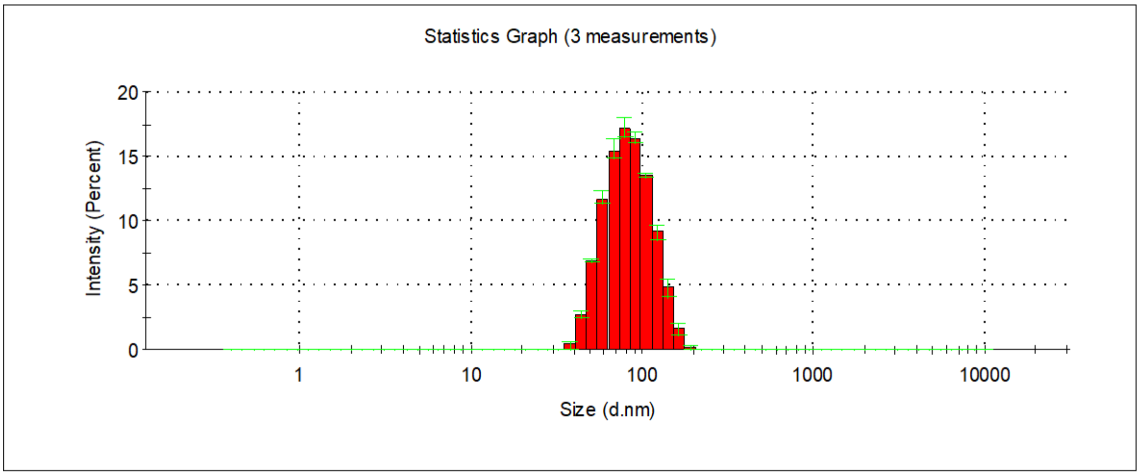

C

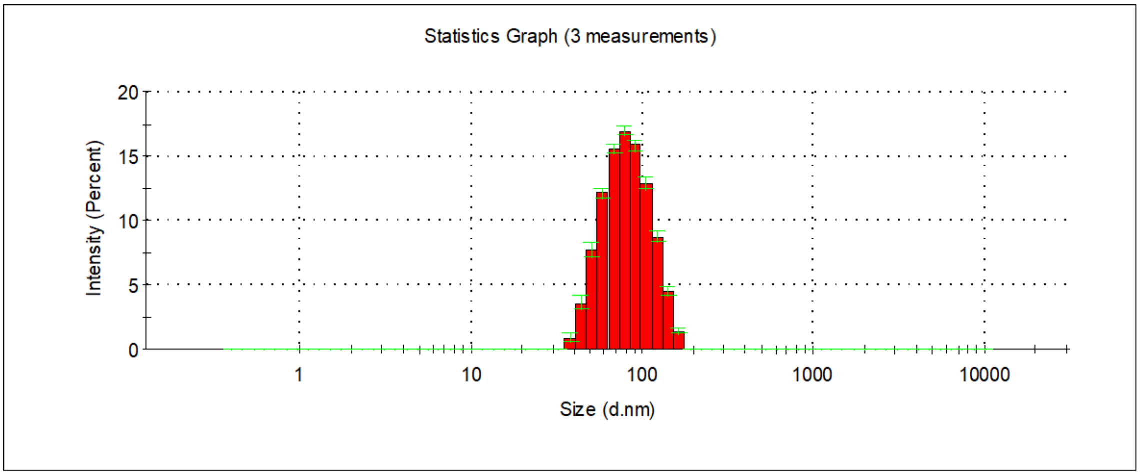

D

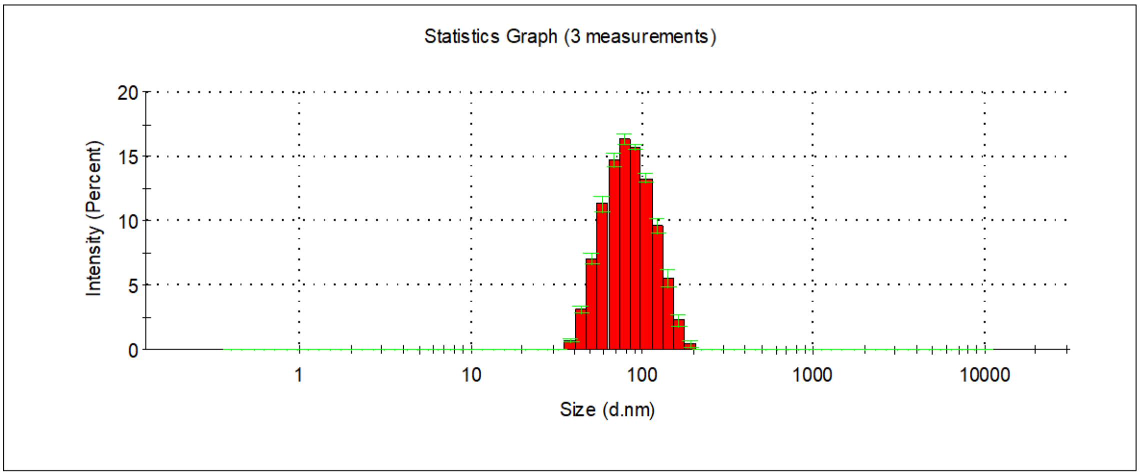

Figure S1. Micellar size and size distribution (PDI) of free and drug-loaded Soluplus® micelles (5% w/v) at 25 °C. A) Soluplus® (5% w/v) micelles. B) Histamine (5 mg/mL)-loaded Soluplus® (5% w/v) micelles. C) Paclitaxel (4 mg/mL)-loaded Soluplus® (5% w/v) micelles. D) Histamine (5 mg/mL) and Paclitaxel (4 mg/mL)-loaded Soluplus® (5% w/v) micelles. Data are expressed as mean ± S.D. (n = 3).

Figure S2

A

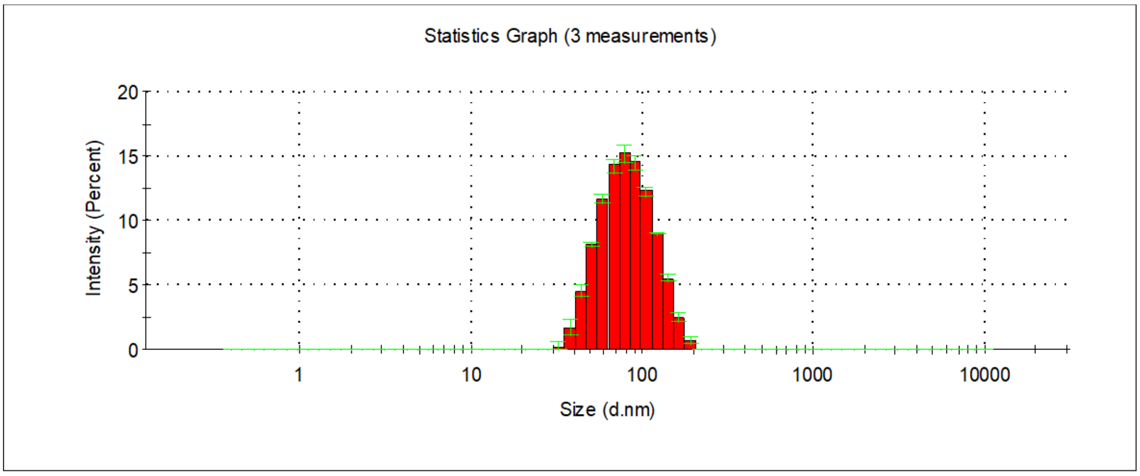

B

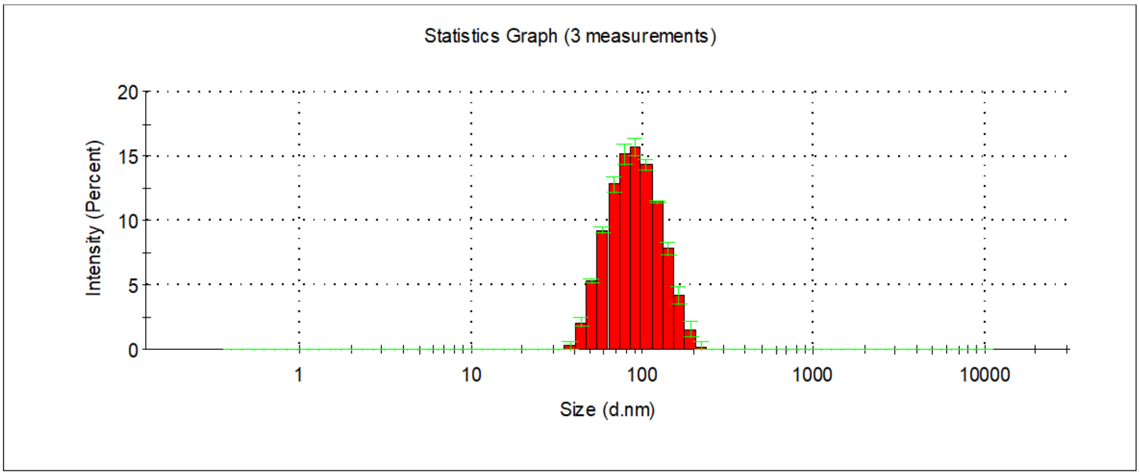

C

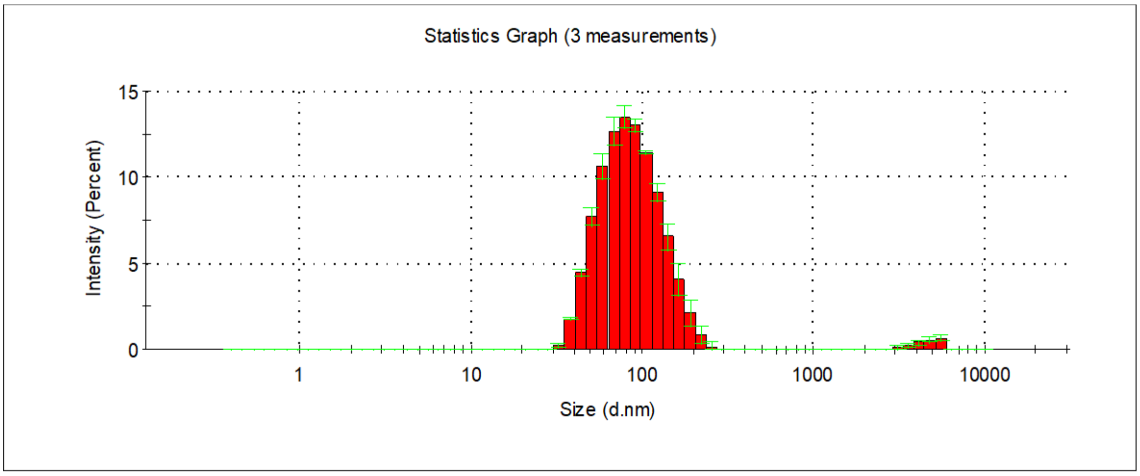

D

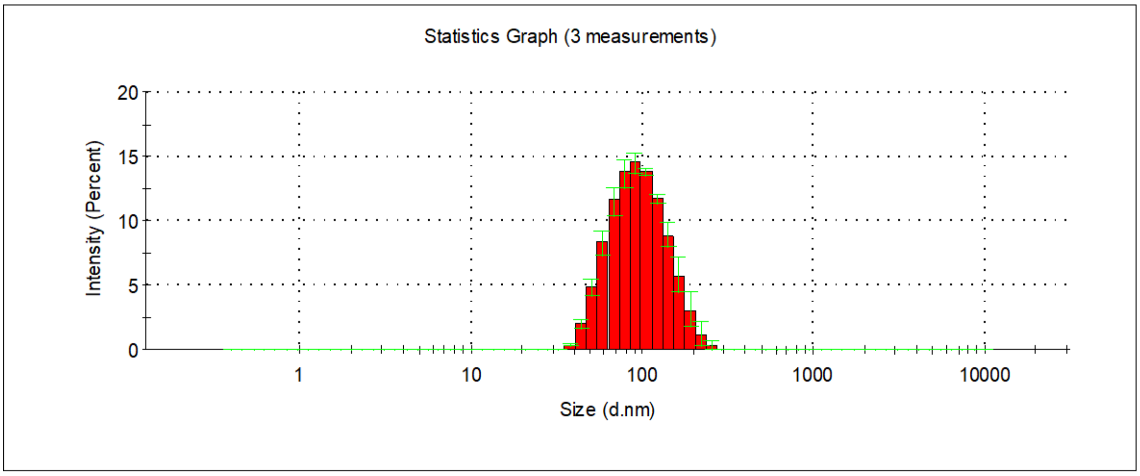

Figure S2. Micellar size and size distribution (PDI) of free and drug-loaded Glucose-Soluplus® micelles (5% w/v) at 25 °C. A) Glucose-Soluplus® (5% w/v) micelles. B) Histamine (5 mg/mL)-loaded Glucose-Soluplus® (5% w/v) micelles. C) Paclitaxel (4 mg/mL)-loaded Glucose-Soluplus® (5% w/v) micelles. D) Histamine (5 mg/mL) and Paclitaxel (4 mg/mL)-loaded Glucose-Soluplus® (5% w/v) micelles. Data are expressed as mean ± S.D. (n = 3).

Figure S3

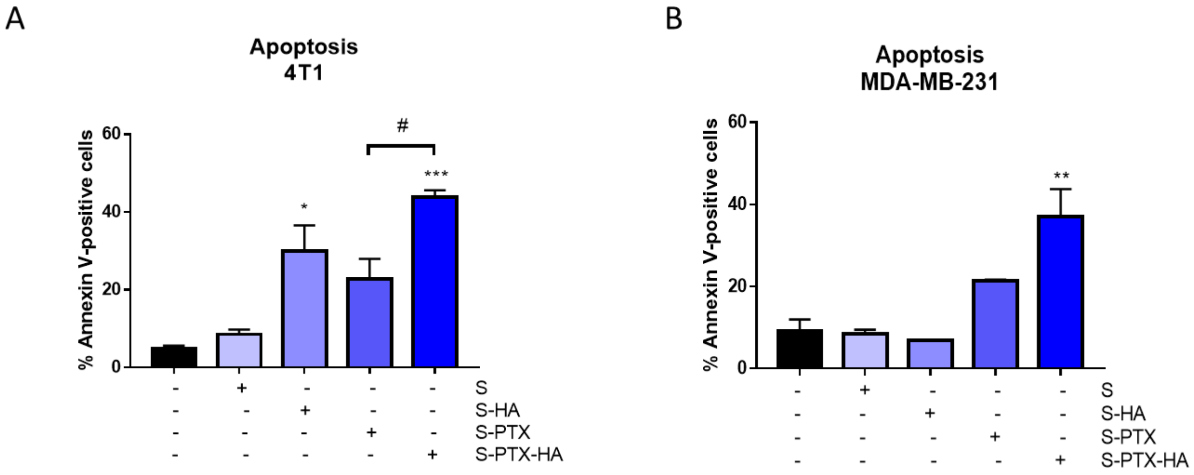

Figure S3. Modulation of apoptosis in 4T1 and MDA-MB-231 cells by nanomicellar formulations. A) 4T1 and B) MDA-MB-231 cells were treated with micellar systems (PTX 0.1 μg/mL) for 72 h. The percentage of Annexin-V was evaluated by flow cytometry. Error bars represent the mean ± SEM (n = 3 independent experiments performed in triplicates). (ANOVA, Tukey's multiple comparisons test, *P<0.05, **P<0.01, ***P<0.001 vs. control, #P<0.05).

Figure S4

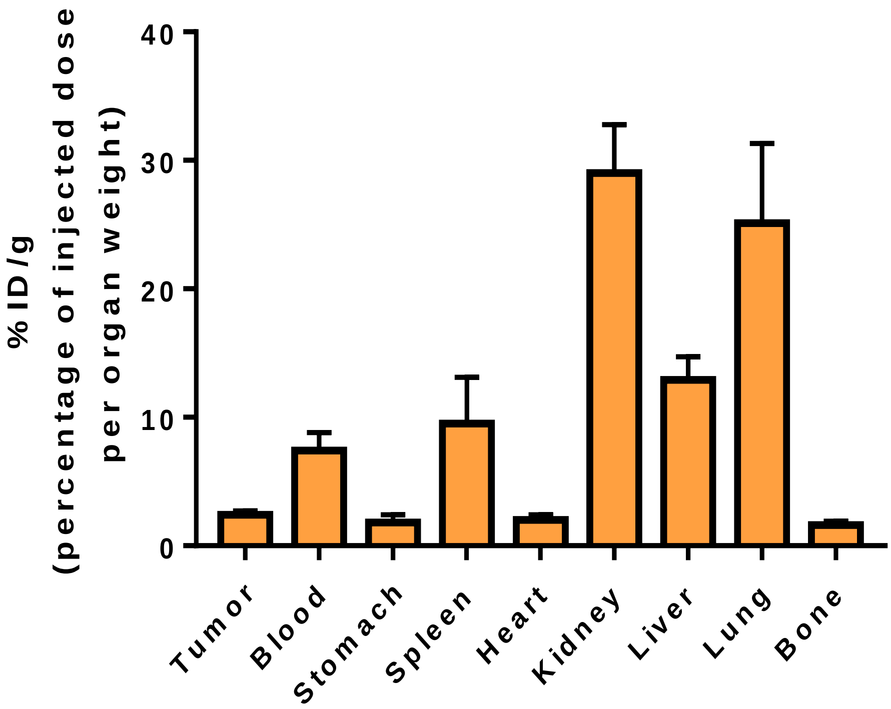

Figure S4. ^{99m}Tc-radiolabeled micelles bio-distribution in 4T1 TNBC model developed in BALB/c mice. Animals were anesthetized with isoflurane 2%v/v and O_2 as carrier. ^{99m}Tc-radiolabeled GS micelles were i.v. administered (tail vein) in 0.05–0.1mL (3.7–37 MBq or 0.1–1 mCi/animal) to 4T1 tumor-bearing animals. An *ex vivo* study was performed to evaluate biodistribution of radioactive 1 h after inoculation. Mice were euthanized in a CO_2 chamber, and organs of interest were excised, weighed, and radioactivity was measured in a well-type solid scintillation counter (Alfanuclear, ZX, Argentina) to express results as percentage of injected radioactive dose normalized by the organ weight (%ID/g). Bars represent mean ± SD (n=4 animals per group).

Figure S5

A

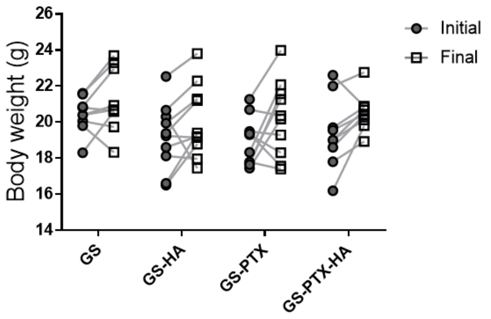

B

Experimental group	*Tissue weight relative to body weight (mean ± SEM)*		
	**Heart**	**Liver**	**Spleen**
**GS**	0.006 ± 0.001	0.070 ± 0.001	0.014 ± 0.002
**GS-HA**	0.006 ± 0.001	0.065 ± 0.002	0.012 ± 0.001
**GS-PTX**	0.007 ± 0.001	0.064 ± 0.001	0.014 ± 0.001
**GS-PTX-HA**	0.007 ±0.001	0.060 ± 0.001	0.015 ± 0.001

C

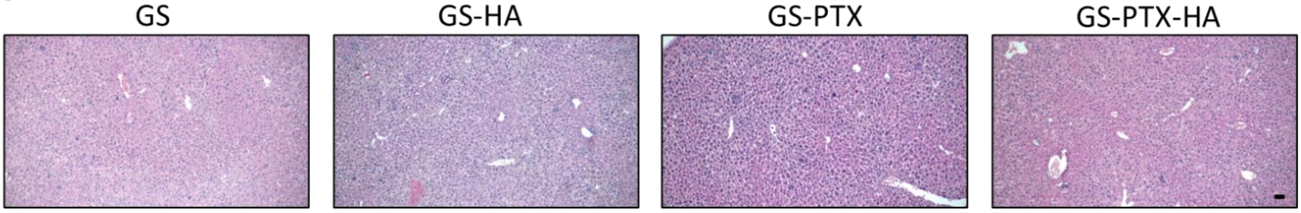

Figure S5. A) Initial and final body weights. B) The weight of heart, liver, and spleen relative to final body weight of 4T1 tumor-bearing mice are shown (n= 9-10). Data represent the mean ± SEM. **C)** Representative images of the liver samples stained with hematoxylin & eosin. x100 original magnification. Scale bar = 20 µm.
